# Associations Between Sad Feelings and Suicide Behaviors in the 2019 Youth Risk Behavior Survey: A Call for Action

**DOI:** 10.3389/fped.2021.694819

**Published:** 2021-09-14

**Authors:** Deana G. Trimble, Aruna Chandran

**Affiliations:** ^1^Department of Health, Behavior, and Society, Johns Hopkins Bloomberg School of Public Health, Institute for Global Tobacco Control, Baltimore, MD, United States; ^2^Department of Epidemiology, Johns Hopkins Bloomberg School of Public Health, Baltimore, MD, United States

**Keywords:** YRBS, youth, suicide, suicidal behaviors, mental health, sad feelings

## Abstract

**Purpose:** Suicide is the second leading cause of death among high school aged students in the United States. Significant risk factors for suicidal behaviors among youth include diagnoses of depression or other mental illnesses. The association between self-reported sad feelings and suicidal behaviors has been understudied in the literature among United States high school students.

**Methods:** The 2019 National Youth Risk Behavior Survey (YRBS) school-based questionnaire, coordinated by the CDC, captured a nationally-representative sample (*N* = 13,677) of students' responses to health-related behaviors. National sex-stratified prevalences of sad feelings and suicidal behaviors were calculated. Adjusted weighted logistic regression was used to examine the association between self-reported sad feelings and suicidal behaviors.

**Results:** Out of 13,677 high-school students, 35.8% of students self-reported sad feelings. Suicidal behaviors in the overall sample included 18.2% had seriously considered suicide, 15.2% made a plan on how they would attempt suicide, and 7.3% attempted suicide within the past 12 months. There was an 8–11-fold increased odds of all suicidal behaviors among those who reported sad feelings among both females and males.

**Conclusions:** This study reveals a remarkably high prevalence of sad feelings among US youth, and underscores a significant association between self-reported sad feelings and suicidal behaviors among this population. The YRBS survey, routinely administered across US high school students, should be better leveraged to target interventions toward these high-risk youth in order to decrease the significant burden of suicidal behaviors among adolescents.

## Implications and Contribution

This study highlights the high prevalence of sad feelings among US adolescents, and a notably strong association between sad feelings and suicidal behaviors in this population. The YRBS survey should be leveraged to target interventions toward this high-risk population in order to decrease the burden of suicide among US adolescents.

## Background

Suicide is the tenth leading cause of death in the United States, responsible for over 48,000 deaths annually (1, 2). Suicide mortality is particularly concerning among teenagers and young adults in the United States, with 6,211 deaths in 2018 among individuals 15–24 years of age, making it the second leading cause of death in this age group (2). The broader construct of suicidal behaviors encompasses suicide ideation (thoughts about suicide), attempted suicide (intentional self-harm with the goal of taking one's own life), and completed suicide (intentional self-harm resulting in death) (3). The 2019 Center for Disease Control's Youth Risk Behavior Survey (YRBS) showed a prevalence of suicide ideation among high school students of 18.8%, and increase from 17.2% in 2017 (4). Similarly, the prevalence of attempted suicide among high school students was 8.9%, an increase from 7.4% in 2017 (4). Increased prevalence of suicide ideation and attempts was noted among individuals reporting female sex, American Indian/Alaska Native, Black/African American or Mixed race, and gay/lesbian/bisexual orientation (4).

Many studies have shown mental health illnesses to be a significant risk factor for suicidal behaviors; up to 90% of adolescents who have died by suicide have a reported history of mental illness (5–8). Among adolescents, depression is the mental health diagnosis most strongly associated with suicide (5, 8). While the YRBS survey does not ask about diagnosis of depression, it does asks youth to report feelings of sadness or hopelessness (hereafter referred to as “sad feelings”) that persist for 2 weeks. Importantly, persistent sad feelings are consistent with some of the DSM-5 diagnostic criteria for depression (9). To our knowledge, there have not been any evaluations within the YRBS of the association between self-reported sad feelings and suicidal behaviors. If self-reported sad feelings are significantly associated with suicidal behaviors, this represents a unique opportunity to identify teens that are potentially at high risk for suicidal behaviors through a survey that is routinely administered in middle and high schools across the US. Therefore, in this study we aim to quantify the association between sad feelings and suicidal behaviors using the national 2019 YRBS survey.

## Methods

### Data Source

This study utilizes data from the 2019 Youth Risk Behavior Surveillance System (YRBSS) survey data, a nationally-representative survey coordinated by the Centers for Disease Control and Prevention (CDC) to monitor health-related behaviors of middle and high school students in the United States (10). The YRBSS has collected data since 1991 on a biennial basis and aims to track the health behaviors that contribute to the leading causes of death, disability, and social problems among United States youth (10). The data for the YRBSS is collected using anonymous self-administered questionnaires completed in school (11). Analytic weights are provided in the YRBS dataset in order to allow for inferences about the broader United States population (11).

The final sample for the 2019 data were 13,677 questionnaires collected from 136 schools across the United States (11). The response rates for the data include a 75.1% school response rate, 80.3% student response rate, and 60.3% overall response rate (11).

### Measures

Sad feelings were captured using the question: “During the past 12 months, did you ever feel so sad or hopeless almost every day for 2 weeks or more in a row that you stopped doing some usual activities?” (Yes/No). Suicidal behaviors were captured across three questions: “During the past 12 months, did you ever seriously consider attempting suicide?” (considered suicide), “During the past 12 months, did you make a plan about how you would attempt suicide?” (made a suicide plan), and “During the past 12 months, how many times did you actually attempt suicide?” (attempted suicide).

Covariates included age, sex, race/ethnicity, sexual orientation, school-based bullying and cyber-bullying. Age was asked “How old are you?” (integer values from 12 or younger to 18 or older). Sex was asked “What is your sex?” (female or male). Race/ethnicity was asked as “What is your race” and “Are you Hispanic or Latino?”, and recoded for responses (American Indian/Alaska Native, Asian, Black or African American, Native Hawaiian/Other Pacific Islander, White, Hispanic/Latino, Multiple-Hispanic, or Multiple-Non-Hispanic). Sexual orientation was captured as “Which of the following best describes you?” (Heterosexual/straight, or Gay/lesbian/bisexual/not sure). School-based bullying was captured as “During the past 12 months, have you ever been bullied on school property?” and Cyber-bullying as “During the past 12 months, have you ever been electronically bullied? (Count being bullied through texting, Instagram, Facebook, or other social media.)”. Substance use behaviors were captured in ever-use questions for each of the following substances: cigarette, electronic vapor product, alcohol, marijuana, synthetic marijuana, prescription pain medicine, cocaine, inhalants, heroin, methamphetamines, ecstasy, hallucinogenic drugs, and steroids. For this analysis, reported use of any of these substances was coded as “ever use.”

### Statistical Analysis

Descriptive statistics showing frequencies of core sociodemographic variables for the overall population, and those reporting sad feelings, considered suicide, made a suicide plan, and attempted suicide were generated using weighted data. Frequencies were also stratified by sex, given the significant differences in prevalence of suicide behaviors by sex. Associations between sad feelings and each of the suicidal behaviors stratified by sex were assessed using weighted logistic regression models adjusted for the above-listed covariates. All data analysis was performed using Stata 16 (StataCorp, College Station, TX).

## Results

### Sample Demographics

The dataset included a total unweighted sample of 13,677 high school students (Table 1). The age distribution ranged from age 12 and under to age 18 and older, with most of the students (85.4%) falling into the 14–17 age range. The students were similarly divided between grade levels, with 26.4% in 9th grade, 25.3% in 10th grade, 24.1% in 11th grade, 23.4% in 12th grade, and 0.2% in an ungraded or other grade. Approximately half the students were female (48.9%) and half were male (50.2%). The race/ethnicity distribution of students included 49.6% white (non-Hispanic), 25.3% Hispanic/Latino, 11.8% Black or African American (non-Hispanic), 4.9% Asian (non-Hispanic), 0.6% American Indian or Alaska Native (non-Hispanic), 0.3% Native Hawaiian or other Pacific Islander (non-Hispanic), and 20.7% multiple race (non-Hispanic) students. The majority (79.2%) of students identified as heterosexual, and 14.7% identified as gay or lesbian, bisexual, or unsure of their sexual identity. Among all student respondents, 51.5% reported ever tobacco use, 54.7% reported ever alcohol use, and 40.1% reported ever illicit drug use (including marijuana). Lastly, 19.3% of students reported they were bullied at school and 15.5% reported having been bullied electronically.

**Table 1:** Descriptive statistics (weighted counts and percent) of high school students in the YRBS 2019.

	**Total sample** **(*N* = 13,677)**	**Sad feelings^a^** **(*N* = 4,953)**	**Suicidal behaviors**		
			**Considered suicide^b^** **(*N* = 2,527)**	**Made a suicide plan^c^** **(*N* = 2,117)**	**Attempted suicide^d^** **(*N* = 1,018)**
**Age (years)**						
12 or younger	41 (0.3)	23 (0.5)	15 (0.6)	16 (0.8)	11 (1.1)
13	13 (0.1)	4 (0.1)	1 (<0.1)	2 (0.1)	2 (0.2)
14	1,617 (11.8)	543 (11.0)	287 (11.4)	233 (11.0)	111 (10.9)
15	3,369 (24.6)	1,128 (22.8)	596 (23.6)	509 (24.0)	259 (25.4)
16	3,480 (25.5)	1,311 (26.5)	663 (26.3)	556 (26.2)	252 (24.8)
17	3.218 (23.5)	1,201 (24.2)	618 (24.4)	516 (24.4)	243 (23.9)
18 or older	1,864 (13.6)	719 (14.5)	334 (13.2)	275 (13.0)	135 (13.3)
Missing	76 (0.6)	22 (0.4)	13 (0.5)	11 (0.5)	5 (0.5)
**Grade level**						
9th	3,614 (26.4)	1,184 (23.9)	627 (24.8)	526 (24.9)	284 (27.9)
10th	3,460 (25.3)	1,261 (25.5)	630 (24.9)	525 (24.8)	255 (25.1)
11th	3,291 (24.1)	1,234 (24.9)	628 (24.9)	533 (25.2)	236 (23.2)
12th	3,197 (23.4)	1,233 (24.9)	620 (24.5)	510 (24.1)	228 (22.4)
Ungraded or other	24 (0.2)	8 (0.2)	4 (0.1)	4 (0.2)	5 (0.5)
Missing	91 (0.7)	33 (0.7)	18 (0.7)	20 (0.9)	10 (1.0)
**Sex**						
Female	6,690 (48.9)	3,086 (62.3)	1,591 (62.9)	1,317 (62.2)	627 (61.6)
Male	6,862 (50.2)	1,811 (36.6)	900 (35.6)	766 (36.2)	375 (36.9)
Missing	125 (0.9)	56 (1.1)	37 (0.1)	34 (1.6)	15 (1.5)
**Race/Ethnicity**						
White, NH	6,784 (49.6)	2,422 (48.9)	1,282 (50.7)	1,051 (49.7)	481 (47.3)
Black or African American, NH	1,617 (11.8)	497 (10.0)	268 (10.6)	237 (11.2)	121 (11.8)
Hispanic/Latino	3,461 (25.3)	1,363 (27.5)	589 (23.3)	504 (23.8)	254 (24.9)
Asian, NH	671 (4.9)	210 (4.2)	131 (5.2)	107 (5.1)	43 (4.2)
American Indian or Alaska Native, NH	86 (0.6)	38 (0.8)	29 (1.2)	20 (1.0)	16 (1.5)
Native Hawaiian/Pacific Islander, NH	44 (0.3)	16 (0.3)	7 (0.3)	6 (0.3)	3 (0.3)
Multiple races, NH	2,836 (20.7)	265 (5.4)	151 (6.0)	131 (6.2)	66 (6.4)
Missing	423 (3.1)	141 (2.8)	71 (2.8)	60 (2.8)	36 (3.5)
**Sexual orientation**						
Heterosexual	10,831 (79.2)	3,457 (69.8)	1,554 (61.5)	1,293 (61.1)	583 (57.2)
Gay or Lesbian	321 (2.3)	188 (3.8)	129 (5.1)	103 (4.8)	54 (5.3)
Bisexual	1,115 (8.2)	743 (15.0)	529 (20.9)	462 (21.8)	235 (23.1)
Not sure of identity	573 (4.2)	266 (5.4)	169 (6.7)	134 (6.3)	71 (7.0)
Missing	837 (6.1)	298 (6.0)	147 (5.8)	126 (6.0)	75 (7.4)
**Substance use**						
Ever tobacco use	7,043 (51.5)	3,179 (64.2)	1,739 (68.8)	1,447 (68.3)	733 (72.0)
Ever alcohol use	7,479 (54.7)	3,318 (67.0)	1,791 (70.9)	1,497 (70.7)	751 (73.8)
Ever illicit drug use	5,486 (40.1)	2,717 (54.9)	1,571 (62.2)	1,308 (61.8)	711 (69.8)
**Bullying**						
Ever bullied at school	2,636 (19.3)	1,618 (32.7)	977 (38.7)	842 (39.8)	458 (45.0)
Ever bullied electronically	2,125 (15.5)	1,369 (27.6)	830 (32.8)	710 (33.5)	400 (39.3)

a *Felt sad or hopeless for two consecutive weeks*.

b *Seriously consider attempting suicide during the past 12 months*.

c *Made a plan about how they would attempt suicide in the past 12 months*.

d *Actually attempted suicide in the past 12 months*.

### Sex-Stratified Prevalence of Sad Feelings and Suicidal Behaviors

Overall, 35.8% of students reported feeling sad almost every day for 2 consecutive weeks or more in a row that stopped them from doing some usual activities (Table 2). Females reported higher prevalence of sad feelings than males (46.1 vs. 26.4%).

**Table 2:** Sex stratified prevalence (weighted counts and percent) of sad feelings, suicidal behaviors, and substance use behaviors among high school students in the YRBS 2019 (*N* = 13,677).

	**Females number (%)**	**Males number (%)**	**Pearson's chi-square**
**Sad feelings**	3,086 (46.1)	1,811 (26.4)	<0.001
**Suicidal behaviors**			
Considered suicide	1,591 (23.8)	900 (13.1)	<0.001
Made a suicide plan	1,317 (19.7)	766 (11.2)	<0.001
Attempted suicide	627 (9.4)	375 (5.5)	<0.001
**Substance use behavior**			
Ever tobacco use	3,469 (51.8)	3,516 (51.2)	0.700
Ever alcohol use	3,907 (58.4)	3,507 (51.1)	<0.001
Ever other drug use	2,693 (40.3)	2,726 (39.7)	0.732

Nearly one-fifth of students (18.2%) reported they seriously considered suicide in the past 12 months. Stratum-specific results showed more females considered suicide (23.8%) than males (13.1%). Comparing all students, 15.2 percent made a plan about how they would attempt suicide in the past 12 months. More females (19.7%) than males (11.2%) reported that they made a plan about how they would attempt suicide. Lastly, 7.3 percent reported they actually attempted suicide in the past 12 months. Among those who actually attempted suicide, there were more females (9.4%) than males (5.5%).

Of those who reported sad feelings or any of the suicidal behaviors, approximately two-thirds (63%) were female and one-third (36%) were male. Of those that reported substance use behavior, the prevalence was nearly 50% female and 50% male for tobacco use, alcohol use, and other drug use.

### Association Between Sad Feelings and Suicidal Behaviors

All suicidal behaviors were significantly associated with sad feelings for both females and males (Table 3). Increases in sad feelings were associated with a nearly 10-fold increased odds of seriously considering suicide [adjusted Odds Ratio (aOR): 9.91, 95% Confidence Interval (CI): 7.6, 13.0] among females, and an over 11-fold increased odds among males (aOR: 11.7, 95% CI: 8.4, 16.2). Increases in sad/hopeless feelings increased the odds of making a suicide plan by 8.17 (95% CI: 6.1, 11.0) among females and by 9.02 (95% CI: 7.0, 11.7) among males. Finally, increases in sad/hopeless feelings increased the odds of suicide attempts by 9.2 (95% CI: 5.2, 16.4) among females and 8.92 (95% CI: 5.1, 15.7) among males. Of note, being bullied (both at school and electronically), substance use, and identifying as a sexual minority were also associated with a significantly increased odds of all suicide outcomes. Age group was not significant in the model, and self-identified racial category was inconsistent in its association with the outcomes.

**Table 3:** Association between feelings of sadness or hopelessness and suicidal behaviors, stratified by sex, among high school students in the YRBS 2019 (*N* = 13,677).

	Adjusted odds ratio^*^ (95% Confidence interval)					
	**Females**			**Males**		
	**Considered suicide**	**Made suicide plan**	**Attempted suicide**	**Considered suicide**	**Made suicide plan**	**Attempted suicide**
**Sad feelings**	**9.91 (7.6, 13.0)**	**8.17 (6.1, 11.0)**	**9.20 (5.2, 16.4)**	**11.7 (8.4, 16.2)**	**9.02 (7.0, 11.7)**	**8.92 (5.1, 15.7)**
**Age (years)**						
13 and younger	Ref	Ref	Ref	Ref	Ref	Ref
14-15	1.29 (0.3, 5.6)	1.04 (0.2, 4.1)	0.09 (0.0, 2.5)	1.94 (0.4, 8.7)	1.11 (0.2, 6.3)	1.67 (0.3, 10.9)
16 and older	1.05 (0.2, 4.4)	0.81 (0.2, 3.2)	0.06 (0.0, 1.8)	2.07 (0.4, 9.7)	1.27 (0.2, 7.7)	2.19 (0.3, 14.0)
**Race**						
White, NH	Ref	Ref	Ref	Ref	Ref	Ref
Black, NH	1.23 (0.9, 1.6)	**1.49 (1.2, 1.9)**	**2.36 (1.6, 3.6)**	0.82 (0.6, 1.2)	0.96 (0.5, 1.7)	1.53 (0.9, 2.4)
Hispanic/Latino	0.87 (0.7, 1.1)	1.03 (0.8, 1.3)	1.35 (0.9, 2.1)	**0.71 (0.5, 0.9)**	**0.69 (0.5, 0.9)**	0.79 (0.5, 1.2)
Other, NH	1.44 (0.9, 2.0)	1.67 (1.1, 2.4)	1.43 (0.9, 2.4)	**1.82 (1.3, 2.5)**	1.2 (0.8, 1.8)	1.62 (0.9, 2.7)
Multiple races, NH	**1.59 (1.2, 2.2)**	**1.71 (1.1, 2.7)**	**2.21 (1.3, 3.7)**	0.97 (0.6, 1.5)	0.97 (0.7, 1.4)	0.91 (0.4, 1.9)
**Sexual orientation**						
Heterosexual	Ref	Ref	Ref	Ref	Ref	Ref
Gay/Lesbian/Bisexual/Unsure	**2.77 (2.3, 3.4)**	**2.72 (2.2, 3.3)**	**2.07 (1.6, 2.7)**	**2.80 (2.0, 4.0)**	**2.22 (1.6, 3.0)**	**3.14 (2.2, 4.5)**
**Substance use**	**2.34 (1.7, 3.2)**	**2.08 (1.6, 2.7)**	**2.44 (1.7, 3.6)**	**1.89 (1.4, 2.5)**	**1.70 (1.3, 2.2)**	**1.65 (1.2, 2.3)**
**Bullying**						
Ever bullied at school	**1.65 (1.3, 2.1)**	**1.55 (1.2, 2.0)**	**1.74 (1.4, 2.2)**	**1.82 (1.2, 2.7)**	**1.77 (1.3, 2.5)**	**1.76 (1.1, 2.9)**
Ever bullied electronically	**1.33 (1.1, 1.6)**	**1.45 (1.1, 1.9)**	**1.56 (1.2, 2.1)**	**1.45 (1.0, 2.0)**	**1.49 (1.0, 2.1)**	**1.86 (1.2, 2.9)**

* *Adjusted for age, sexual orientation, race/ethnicity, substance use, bullying at school and electronic bullying*.

*Bold denotes statistically significant associations (p < 0.05)*.

## Discussion

The increasing rates of suicide among United States high school students raises major concern as it continues to remain the second leading cause of suicide in this age group (2, 12). The results of this study highlight the magnitude of the problem of suicide in United States as nearly one-fifth (18.2%) of the sample reported seriously considering suicide and 7.3% reported making a suicide attempt. This result is on par with the CDC's Web-based Injury Statistics Query and Reporting System (WISQARS) results for non-fatal injuries, which estimated 5.3% of non-fatal injuries for high school-aged youth were caused by self-harm in 2019 (13).

Understanding the potential factors that contribute to this problem is crucial to reducing the rates of these suicidal behaviors. Our study points out a 35.8% prevalence of sad feelings among high school students in the US in 2019. This is significantly higher than the 13.3% prevalence of major depressive episodes among adolescents aged 12–17 (14). Importantly, we show large (between 8 and 11-fold increases) statistically significant positive associations between self-reported sad feelings and suicidal behaviors among both female and male high school students in the United States. These findings emphasize previous research in demonstrating the relationship between diagnosed mental illness and suicidal behaviors (5–7), and provide unique insight on the impact of self-reported feelings of sadness or hopelessness. These unique findings highlight the need for action as the magnitude of the association between sad feelings and suicidal behaviors is high, regardless of formal mental health diagnoses. Since students readily indicated their feelings in this survey, there is a clear need for help in this demographic.

Previous research examining sex differences in suicidal behavior in this population has shown that females are more likely to engage in suicidal behaviors, but males have higher suicide mortality (15, 16). Our study underscores this in that more females reported suicide ideation, suicide planning and attempted suicide. Notably, we show that the relationship between sad feelings and suicide ideation, planning and attempted suicide was actually higher for males. This association is important given what is known about sex differences in suicide behaviors; our study suggests that associations between sad feelings and suicide outcomes are similar among male and female youth. This suggests that interventions to promote discussions about sad feelings should be prioritized among both males and females.

This study presents several limitations. Firstly, the data used in this study from the 2019 YRBS was cross-sectional; therefore, we cannot draw conclusions about the causal link between sad feelings and suicidal behaviors. Second, this survey relies on self-report measures from adolescents, which could result in under- or over-reporting bias especially for those questions pertaining to sad feelings and suicidal behaviors. Finally, the YRBS questionnaire does not include questions for two important potential confounders. The questionnaire used has no measure of socioeconomic status, which has been associated with suicidal behaviors among youth (17). Additionally, the questionnaire only asks for the students' sex, and does not ask about gender identity, which has also been associated with suicidal behaviors among youth (18).

Our study strengthens existing research connecting sad or hopeless feelings to suicidal behaviors. With such a high prevalence of sad feelings among US high school students and a remarkably high magnitude of the relationship between sad feelings and suicidal behaviors, these study findings underscore the need for action in this demographic. The YRBS is performed nationally every 2 years, providing every local jurisdiction with school-level data on feelings of sadness and suicidal behaviors. These data can then inform targeted interventions for students experiencing these feelings which could ultimately decrease the number of suicidal behaviors in this population. We call on all local jurisdictions to leverage this routinely collected data to better address poor mental health among US teens.

## Data Availability Statement

Publicly available datasets were analyzed in this study. This data can be found here: https://www.cdc.gov/healthyyouth/data/yrbs/data.htm; https://www.cdc.gov/healthyyouth/data/yrbs/files/2019/XXH2019_YRBS_Data.dat.

## Author Contributions

DT: conceptualization, data curation, formal analysis, investigation, methodology, project administration, writing the original draft, and reviewing and editing. AC: data curation, formal analysis, methodology, project administration, supervision, and reviewing and editing. All authors contributed to the article and approved the submitted version.

## Conflict of Interest

The authors declare that the research was conducted in the absence of any commercial or financial relationships that could be construed as a potential conflict of interest.

## Publisher's Note

All claims expressed in this article are solely those of the authors and do not necessarily represent those of their affiliated organizations, or those of the publisher, the editors and the reviewers. Any product that may be evaluated in this article, or claim that may be made by its manufacturer, is not guaranteed or endorsed by the publisher.
